# Biological and molecular studies on specific immune cells treated with checkpoint inhibitors for the thera-personal approach of breast cancer patients (*ex-vivo* study)

**DOI:** 10.32604/or.2022.025249

**Published:** 2022-10-10

**Authors:** MOTAWA E. EL-HOUSEINI, MOSTAFA S. ARAFAT, AHMED M. EL-HUSSEINY, ISLAM M. KASEM, MAHMOUD M. KAMEL, AHMED H. EL-HABASHY, MEDHAT M. KHAFAGY, ENAS M. RADWAN, MAHA H. HELAL, MONA S. ABDELLATEIF

**Affiliations:** 1 Medical Biochemistry and Molecular Biology, Cancer Biology Department, National Cancer Institute, Cairo University, Cairo, 11976, Egypt; 2 Biotechnology Department, Faculty of Science, Cairo University, Giza, 12613, Egypt; 3 Zoology department, Faculty of Science, Cairo University, Giza, 12613, Egypt; 4 Clinical Pathology Department, National Cancer Institute, Cairo University, Cairo, 11976, Egypt; 5 Department of Pathology, National Cancer Institute, Cairo University, Cairo, 11976, Egypt; 6 Surgical Oncology Department, National Cancer Institute, Cairo University, Cairo, 11976, Egypt; 7 Radio-Diagnosis Department, National Cancer Institute, Cairo University, Cairo, 11976, Egypt

**Keywords:** Immunotherapy, breast cancer, PD1, CTLA4, TIDCs, TILs

## Abstract

Immunotherapy becomes a promising line of treatment for breast cancer (BC) however, its success rate is still limited.

**Methods:**

The study was designed to optimize the condition for producing an effective dendritic cell (DCs) based immunotherapy by using DCs and T lymphocytes together with tumor-infiltrating lymphocytes (TILs) and tumor-infiltrating DCs (TIDCs), treated with anti-PD1 and anti-CTLA4 monoclonal antibodies. This mixture of immune cells was co-cultured with autologous breast cancer cells (BCCs) isolated from 26 BC females.

**Results:**

There was a significant upregulation of CD86 and CD83 on DCs (*p* = 0.001 and 0.017, respectively), similarly upregulation of CD8, CD4 and CD103 on T cells (*p* = 0.031, 0.027, and 0.011, respectively). While there was a significant downregulation of FOXP3 and combined CD25.CD8 expression on regulatory T cells (*p* = 0.014 for both). Increased CD8/Foxp3 ratio (*p* < 0.001) was also observed. CD133, CD34 and CD44 were downregulated on BCCs (*p* = 0.01, 0.021, and 0.015, respectively). There was a significant increase in interferon-γ (IFN-γ, *p* < 0.001), lactate dehydrogenase (LDH, *p* = 0.02), and a significant decrease in vascular endothelial growth factor (VEGF, *p* < 0.001) protein levels. Gene expression of FOXP3 and Programmed cell death ligand 1 (PDL-1) were downregulated in BCCs (*p* < 0.001, for both), similarly cytotoxic T lymphocyte antigen-4 (CTLA4, *p* = 0.02), Programmed cell death 1 (PD-1, *p* < 0.001) and FOXP3 (*p* < 0.001) were significantly downregulated in T cells.

**Conclusion:**

*Ex-vivo* activation of immune cells (DCs, T cells, TIDCs, and TILs) with immune checkpoint inhibitors could produce a potent and effective BC immunotherapy. However, these data should be validated on an experimental animal model to be transferred to the clinical setting.

## Introduction

Breast cancer (BC) is the most commonly diagnosed cancer among females in both developed and developing countries. It is the leading cause of cancer‑related death in women worldwide [1]. Despite the advancement in the diagnosis and treatment strategies for BC, the incidence of relapse and metastasis is significantly increased in up to 30% of the patients, especially in those with triple-negative BC. Accordingly, this leads to poor prognosis and elevated mortality rates [2,3].

Over the last few years, many trials were developed for using dendritic cell-based immunotherapy for the treatment of BC, as there is accumulating evidence suggesting the crosstalk between the breast cancer cells and the immune system [4]. Indeed, the ability of DCs to elicit an effective antitumor immune response depends mainly on the maturation state of the DCs. As they circulate in the blood and have the ability to recognize, take up tumor antigen, and process it. Then they migrate to the draining lymph nodes (LN), where they can activate naïve T cells through antigen presentation, and consequently induce an anti-tumor immune response [5,6]. It had been reported that tumor cells can induce regulatory factors that inhibit DCs maturation and activation as a way of evading immune surveillance. These immature DCs (imDCs) can stimulate T-cell anergy and induce regulatory T cells (Tregs) proliferation. This in turn leads to hindering cytotoxic T cell (CTLs) activation and contributes to tumor progression and spread [7,8]. On the other hand, it was found that Tregs down-regulate B7-molecules (CD80 and CD86) on *ex vivo* co-cultured DCs, and this down-modulation could be inhibited by blocking cytotoxic T lymphocyte antigen-4 (CTLA4) molecules [9].

CTLA4 is a potent immunosuppressive molecule that is normally expressed on the surface of activated T cells and a subset of Tregs [10]. During the early stage of carcinogenesis, CTLA4 is constitutively over-expressed on the Tregs and blocks the interaction between CD28 ligand on T lymphocytes and the CD80/86 co-receptors on DCs. This leads to inhibition of DCs activation, decreased production of IL-12, and suppression of CD8+ CTLs proliferation [11,12]. Accordingly, inhibition of an effective antitumor immune response and induction of tumor immune tolerance will be developed [13].

The Tregs have been reported to be increased in the peripheral blood and the tumor tissue of cancer patients. They are characterized by the expression of the surface markers CD25, CTLA4, CD103, CD45RO, GITR, and the Foxp3 transcription factor [14]. They also secrete IL-10 and TGF-β, which mediate immunosuppression and promotion of tumor growth [15].

Programmed cell death 1 (PD-1), is a receptor expressed on the surface of T, B, and NK cells, which belongs to the B7-CD28 superfamily [16]. It binds to programmed cell death-ligand 1 (PDL1), expressed by activated B cells, T cells, dendritic cells, macrophages, fibroblasts, and tumor cells of many types of solid cancers including breast cancer [17,18]. The binding of PD1 with its ligand PDL1 leads to inducing T-cell apoptosis. Thus, Blockade of the PD-1/PDL1 pathway with specific monoclonal antibodies is considered an important therapeutic approach for activation of the anti-tumor immune response [19].

It had been reported by many recently published studies that breast cancer patients had dysfunctional DCs with decreased MHC Class II and CD86 expression, as well as decreased IL-12 secretion [20,21]. These patients also had a significant decrease of lymphocytes and increased numbers of functionally suppressive CD4+CD25+ Tregs in the peripheral blood and tumor microenvironment, With a subsequent increase of CTLA4 expression on both T cells and breast cancer cells [21–23]. Hence, the aim of the current study is to optimize the conditions for the production of effective DCs-based immunotherapy for those breast cancer patients who have T cell anergy. This will be achieved through ex-vivo activation of DCs and T cells co-cultured with autologous breast cancer cells (BCCs) in the presence of anti-PD1 and anti-CTLA4 monoclonal antibodies. The cell mixture was assessed for activation by comparing different markers before and after interaction with tumor cells including CD86, CD83, and IL12 for assessing the activation of DCs. The levels of CD4, CD8, CD25, CD103, FOXP3, PD1, CTLA4, and IFN-γ for assessing activation of lymphocytes. CD133, CD44, CD34, FOXP3, PDL1, LDH, and VEGF for assessing tumor cell inhibition. We thought this will help for producing a potential active DCs-based immunotherapy that can efficiently eradicate tumor cells, especially in late-stage BC patients.

## Methods

This is a prospective cohort study that included 26 female breast cancer cases who were presented and diagnosed at National Cancer Institute (NCI), Cairo University, during the period from December 2019 to October 2020. All the assessed patients were diagnosed with invasive ductal carcinoma, with the majority of them being stage II; 17/26 (65.4%), six patients (23.1%) were stage III, and only three patients (11.5%) were stage IV. Fifteen patients (57.7%) had Luminal B subtype, and 11 (42.3%) had Luminal A subtype. The assessed BC patients had a median age of 52 with a range from 32 to 76 years old, and they had a mean age of 56 ± 13.9 years old. Patients with positive ER represented 76.9% (20/26), positive PR represented 84.6% (22/26), and those with positive HER2 represented 50% (13/26). Ten patients (38.4) had axillary lymphadenopathy, and 3 patients (11.5%) had distant metastasis.

### Samples

Tumor tissue samples were obtained from each patient during surgery, or through true-cut biopsy in the radio-diagnosis department (NCI). In addition, Peripheral blood (PB) samples (5 ml) were also drawn from each patient in heparinized tubes for isolation of peripheral blood mononuclear cells (PMNCs).

### Preparation of BCCs, TIDCs, and TILs

**Figure 1:**
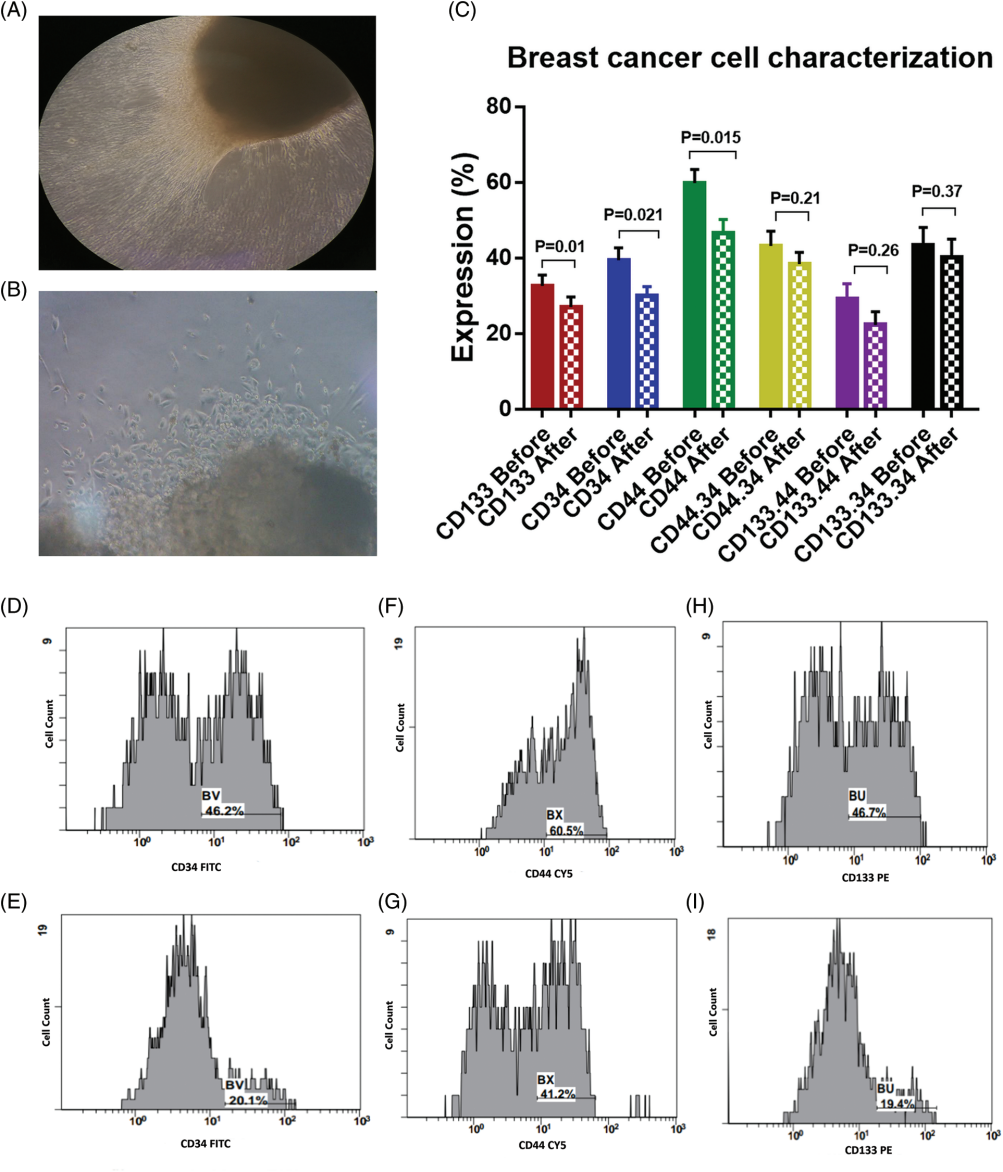
(A) and (B) Outgrowth of tumor tissue explant, with a magnification power for figure A is 10X, and 40X for figure B. (C) Expression levels (mean ± SEM, *n* = 3) of CD133, CD34 and CD44 in breast cancer cells before and after interaction with the dendritic cell-based immunotherapy. Single histogram showing significant decrease in the expression levels of CD34 (D, E), CD44 (F, G) and CD133 (H, I) after interaction with the dendritic cell-based vaccine.

Tumor tissues were cut into 1 × 1 mm pieces and separated into two equal portions: the first portion for the preparation of autologous BCCs, and the second portion for the generation of tumor-infiltrating lymphocytes and DCs (TILs and TIDCs). The autologous BCCs were isolated from the biopsy sample using Collagenase IV according to the manufacturer’s instructions (Gibco-Life Technology, Cat. no. 17104-019). The cells were washed and seeded in DMEM/F12 (GIBCO, life Technology, 41965-039) culture media containing 10% heat-inactivated Fetal bovine serum (FBS), 100 IU penicillin/streptomycin (GIBCO, life Technology), 0.1 mM sodium pyruvate (Lonza, Switzerland), 20 ng/ml insulin-like growth factor (Recombinant Human IGF-1, Gibco-Life Technology, PHG0071), and 20 ng/ml epidermal growth factor (EGF, Gibco-Life Technology, PHG0311). Media was changed every other day till 100% confluence (Figs. 1A, 1B).

The other portion of tumor tissue was digested using collagenase IV (Gibco-Life Technology, 17104-019), DNAse I (Thermo Scientific, EN0525), and Hyaluronidase (SIGMA, H3506-100 MG). The cells were washed with phosphate buffer saline (PBS) and cultured in RPMI-1640 medium (Gibco-Life Technology, 52400-025) supplemented with 10% FBS, penicillin (100 U/ml), and streptomycin (100 μg/ml, all from Gibco-Life Technology), in a humidified incubator at 37°C with 5% CO_2_. The cultured cells were enriched with recombinant human granulocyte monocyte-colony-stimulating factor (GM-CSF; 50 ng/ml, Gibco-Life Technology, Cat. no. PHC2013), interleukin 4 (IL4; 10 ng/ml, Bio Basic Inc-Cat. no. RC212-15), IL12 (20 ng/ml, Bio Basic Inc-Cat. no. RC212-23) and IL2 (4000 IU/ml, Bio Basic Inc-Cat. no. RC212-13), for differentiation and maturation of the TIDCs and TILs [24]. The cells were maintained for 7 days with changing media every other day till mixing with peripheral blood DCs and T cells for further 2 days.

### Preparation of Peripheral Blood Mononuclear Cells (PMNCs)

The PMNCs were isolated from the PB of the patients by Ficoll-Hypaque density gradient centrifugation (Biowest LLC, cat. no. L0560-100, Riverside, MO, USA). Centrifuging was performed at 60–100 xg, for 30–40 min at 18–20°C. The Interphase cells (buffy coat) were drawn and washed twice in PBS without Ca^2+^ and Mg^2+^ (Biowest LLC, Riverside, MO, USA). Then, the isolated cells were cultured in RPMI-1640 medium supplemented with 10% FBS, penicillin (100 U/ml), and streptomycin (100 μg/ml), in a humidified incubator at 37°C with 5% CO_2_. The cultured cells were enriched with 50 ng/ml GM-CSF, 10 ng/ml IL4, 20 ng/ml IL12, and 4000 IU/ml IL2, for differentiation and maturation of the peripheral blood DCs and T cells [25]. The cells were maintained for 7 days with changing media every other day, till mixing with TIDCs and TILs for further 2 days.

### Co-Culture of immune cells (DCs, T cells, TIDCs, and TILs) with BCCs

The mixture of the immune cells was incubated with the BCCs at a ratio of 1:1 in complete culture media RPMI for 24 h. Checkpoint inhibitors including anti-PD1-mAb (5 ng/ml, CD279, eBioscience, cat. no. 16-9985-81), and anti-CTLA4-mAb (5 ng/ml, CD152, Invitrogen, cat. no. 16-1521-81) were added to the mixture for activation of effector immune cells. After that, cells were harvested for immune-phenotyping and molecular assessment, while the media were assessed for cytokine measurement.

### Flow cytometry analysis

The BCCs were assessed for the cell-surface markers CD133, CD34, and CD44 using labeled monoclonal antibodies (PE, FITC, and PerCP-Cy5; respectively, Invitrogen, eBioscience, US) according to the manufacturer’s instructions. Immuno-phenotyping analysis of the tumor cells was performed before and after interacting with the immune cells. Also, Immuno-phenotyping analysis was performed for the immune cells before and after interaction with the BCCs using labeled monoclonal antibodies for the assessment of CD86 (PE, eBioscience, cat. no. 12-0869-42) and CD83 (FITC, EXBIO, cat. no. 1F-677-T100) on DCs, CD8 (PE, BD Biosciences, BD Pharmigen, cat. no. 555367), CD4 (FITC, Immuno Tools, cat. no. 21270043), CD25 (APC, Invitrogen, eBioscience, cat. no. 17-0259-42), FOX-P3 (PE, R&D systems, cat. no. C8214P) and CD103 (APC, Invitrogen, eBioscience, cat. no. 17-1037-42) on lymphocytes, according to the manufactures’ instructions. Isotype control was used to avoid background caused by non-specific binding. Cells were incubated with the specific isotype before analysis, to set the gating regions and distinguish between negative and positive areas. All samples were acquired on multicolour Beckman coulter, Navios flow cytometry (Clare, Ireland, SN: AV47168) using Navios software with a standard 6-colour filter conjugation.

### Molecular assessment using quantitative real‑time PCR (qRt-PCR)

Total RNA was extracted from the isolated immune cells and BCCs using RNeasy extraction blood Mini kit (QIAGEN, cat. no. 74104), and transcribed to cDNA using High-Capacity cDNA Reverse Transcription Kit (Applied Biosystems, cat. no. 4368814) as recommended by the manufacturer’s instructions. The purity and the concentration of the extracted RNA were detected using a spectrophotometer nano-drop (Quawell, Q-500, Scribner, USA). The qRt-PCR was performed using the Quanitech SYBR Green PCR kit (QIAGEN, 204143, CA, USA) in a 25-μl total volume. The mRNA expression was quantified for assessing FOXP3 (Bio Basic Inc.) and PDL1 (Invitrogen) in BCCs, together with FOXP3, PD1, and CTLA4 in the immune cells (all from Invitrogen), primers’ sequences were shown in Table 1. The fluorescence was acquired and detected by (ABI) 7500 fast Real-time PCR system (Applied Biosystems; Thermo Fisher Scientific, Inc., USA). Data were normalized to the housekeeping gene *(β-actin)* using the comparative Ct method (2^−ΔΔCt^) [26].

**Table 1:** The primer sequences of the tested genes

Gene name	Primer sequence
FOXP3	F: CAGCACATTCCCAGAGTTCCTC R: GCGTGTGAACCAGTGGTAGATC
PD1	F: CCAGGATGGTTCTTAGACTCCC R: TTTAGCACGAAGCTCTCCGAT
PDL1	F: TGGCATTTGCTGAACGCATTT R: TGCAGCCAGGTCTAATTGTTT
CTLA4	F: GCCCTGGCACTCTCCTGTTTTT R: GGTTGCCGCACAGACTTCA
B-actin	F: AAAGGGTGGTAACGCAACTA R: GGACCTGACTGACTACCTC

### Protein levels measurement

The harvested media of the cultured cells were assessed before and after the interaction, for measurement of Vascular endothelial growth factor (VEGF), lactate dehydrogenase (LDH), and interferon-gamma (IFN-γ) levels using the ELISA technique. The samples were done in duplicate according to manufacturers’ instructions (Human VEGF-KOMA BIOTECH-cat. no. k0331132, Human IFN- Gamma-KOMA BIOTECH-cat. no. k0331121, and cloud Clone Corp, cat. no. SEB864 Hu for LDH assessment).

### Statistical analysis

Data were analyzed using the Statistical Package of Social Science Software program, SPSS (version 25, Chicago, IL, USA). the data were presented as the mean and standard error of the mean (SEM) for quantitative variables, while as a frequency & percentage for qualitative variables. One-way ANOVA and Paired *T*-test were used for comparison between variables before and after activation. *p* < 0.05 is considered statistically significant. The relative % change was calculated as follows:

Relative % change = [(after activation – before activation)/before activation] * 100.

## Results

### Inhibition of BCCs by the activated immune cell mixture

**Figure 2:**
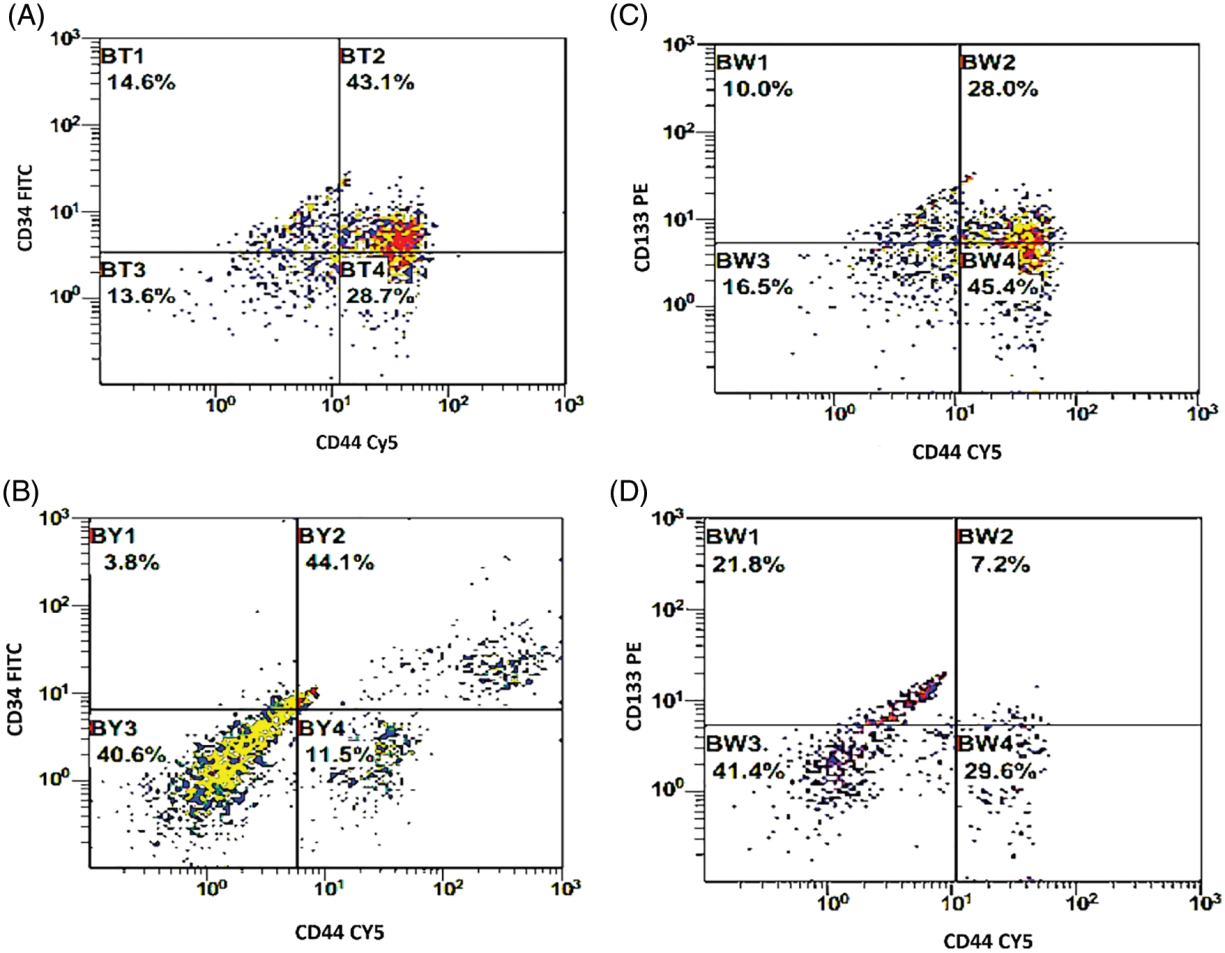
(A, B) Double histogram of breast cancer cells co-expressing both CD34 & CD44 before and after interaction with the dendritic cell-based immunotherapy. (C, D) Double histogram of breast cancer cells co-expressing both CD133 & CD44 before and after interaction with the dendritic cell-based immunotherapy.

The BCCs were examined for the expression of CD133, CD34, and CD44 before and after co-culture with the immune cells which were activated with checkpoint inhibitors (Fig. 1C). There was a significant decrease in the expression level of CD133 by −16.9% [32.6 ± 2.9 and 27.1 ± 2.6; before and after co-culture, respectively, *p* = 0.01]. CD34 was significantly decreased by −23.6% [39.4 ± 3.3 and 30.1 ± 2.4; before and after co-culture, respectively, *p* = 0.021]. Also, CD44 was significantly decreased by −22.2% [59.8 ± 3.7 and 46.5 ± 3.6; before and after co-culture, respectively, *p* = 0.015, Figs. 1D–1I]. In addition, the combined expression of CD44.CD34 was decreased by −10.9% [the expression was 43.1 ± 3.9 before co-culture, compared to 38.4 ± 3.2 after co-culture, *p* = 0.206]. Also, the combined expression of CD133.CD34 was decreased by −7.6% [the expression was 43.4 ± 4.7 before co-culture compared to 40.1 ± 4.8 after co-culture, *p* = 0.368], however, they did not reach a significant level (Fig. 2).

### Activation of the Dendritic Cells (DCs) after priming with BCCs

**Figure 3:**
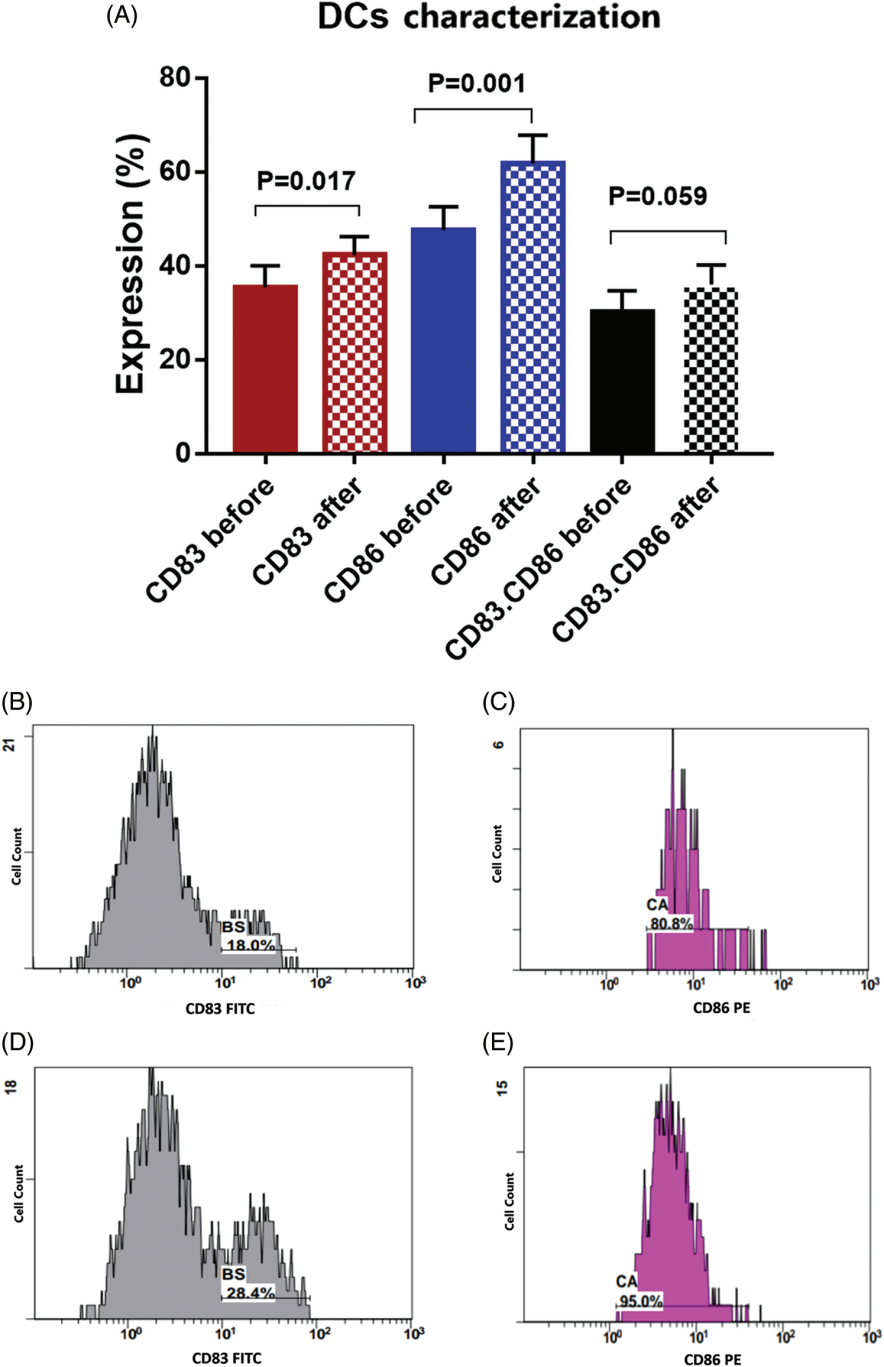
(A) Expression levels (mean ± SEM, *n* = 3) of dendritic cell markers CD83 and CD86 before and after interaction with tumor cells. Single histogram showing significant increase in the expression levels of CD83 (B, D) and CD86 (C, E) after interaction with autologous tumor cells.

There was a significant increase in the expression level of CD83 by 19.2% [35.5 ± 4.6 compared to 42.3 ± 3.9 before and after priming; respectively, *p* = 0.017]. Also, a significant increase in the expression level of CD86 by 29.8% [47.6 ± 4.9 compared to 61.8 ± 6 before and after priming, respectively, *p* = 0.001] was found. While combined expression of CD83. CD86 was 30.1 ± 4.5 before priming, and it was 36 ± 4.26 after priming, with a 19.6% increased percent (*p* = 0.059, Fig. 3).

### Activation of the T cells after interaction with DCs and BCCs

**Figure 4:**
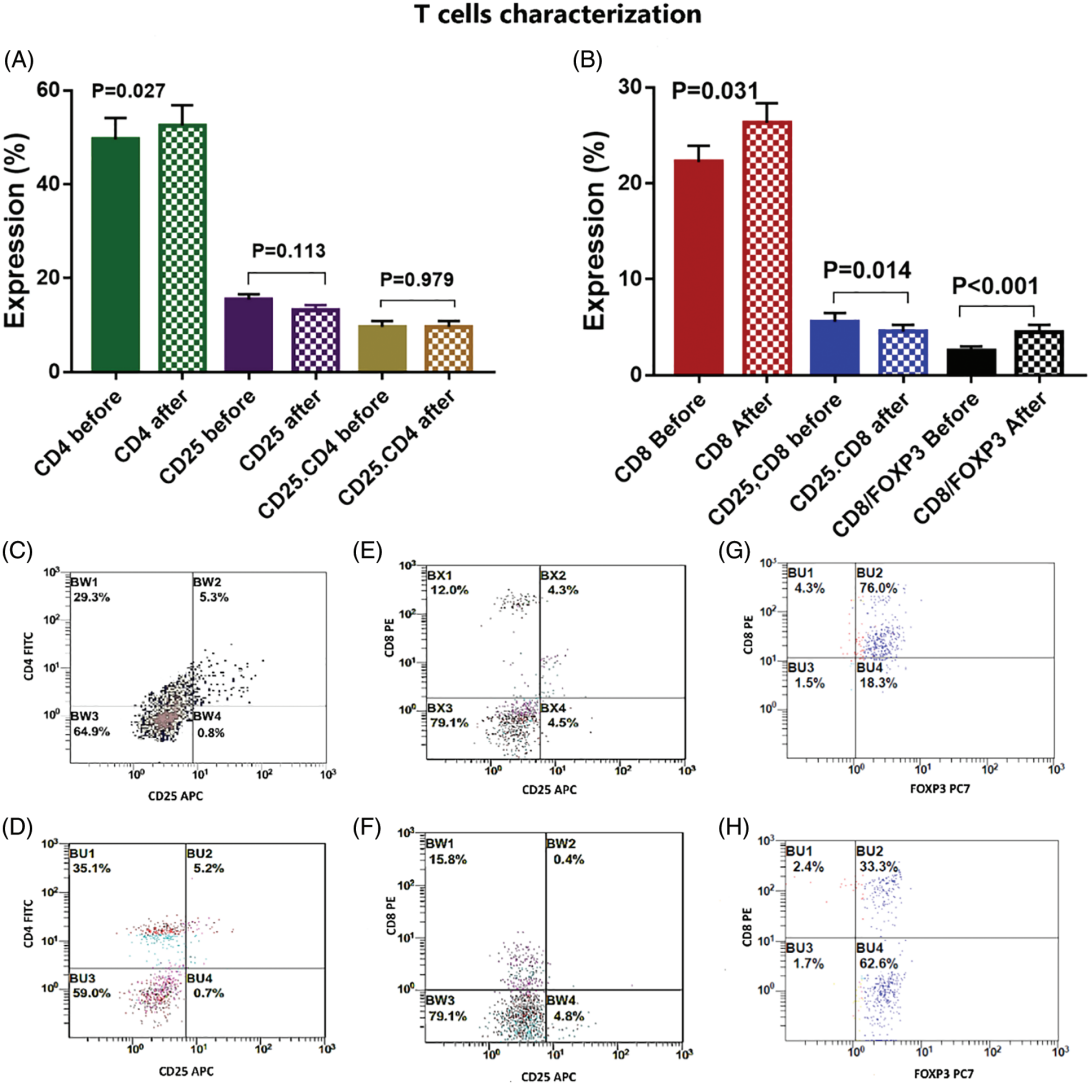
(A) Expression levels (mean ± SEM) of CD4, CD25 and CD4.25 in T cells before and after interaction with autologous tumor cells. (B) Expression levels (mean ± SEM) of CD8, CD8.25 and CD8.FOXP3 in T cells before and after interaction with autologous tumor cells. Double histogram showing decrease of T lymphocytes co-expressing both CD4 & CD25 (C, D), CD8 & CD25 (E, F) and CD8, FOXP3 (G, H) after interaction with autologous tumor cells.

**Figure 5:**
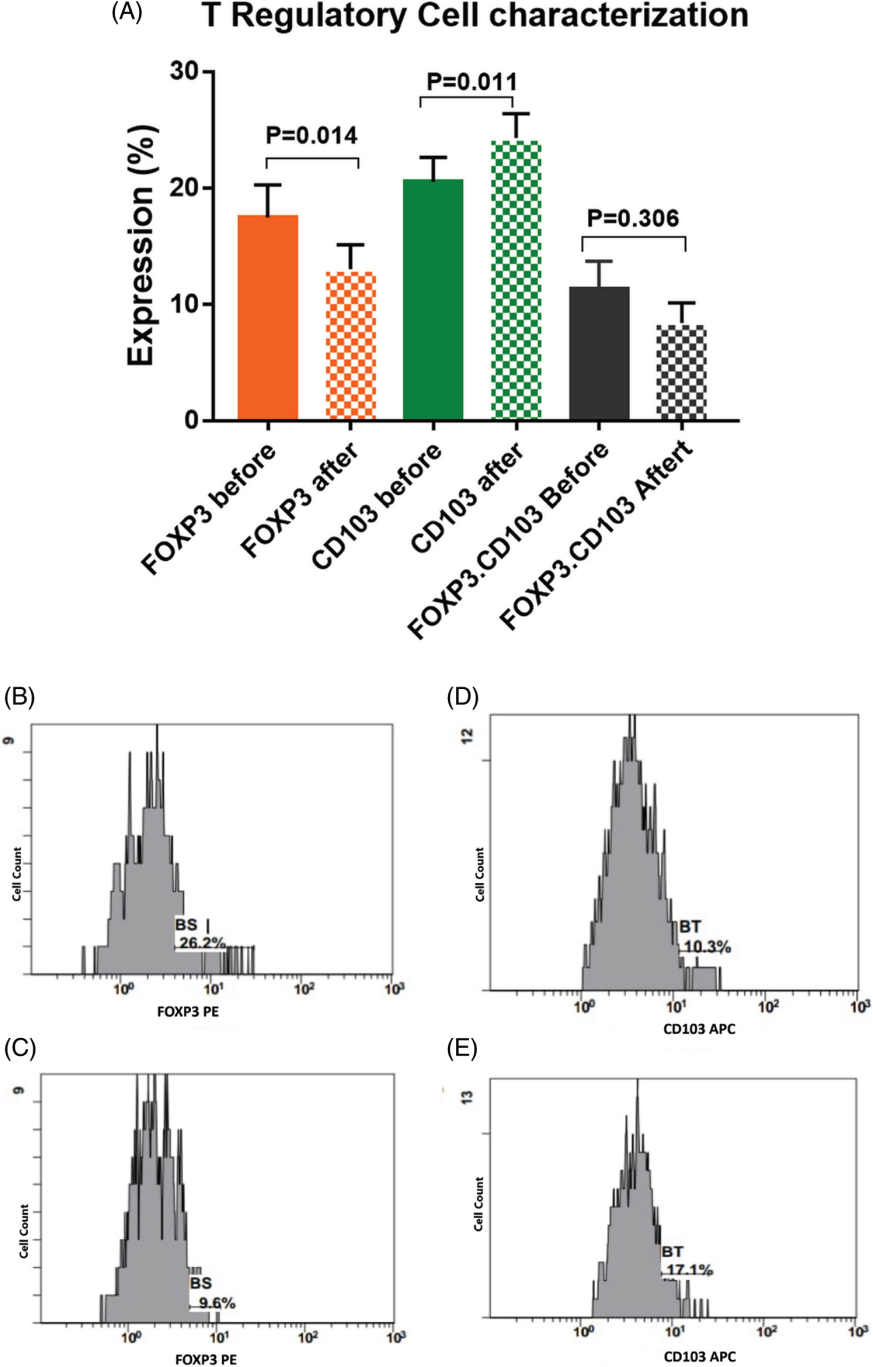
(A) Expression levels (mean ± SEM) of FOXP3, CD103 and CD103.FOXP3 in T cells before and after interaction with autologous tumor cells. Single histogram showing significant decrease in the expression levels of FOXP3 (B, C) and CD103 (D, E) before and after interaction with autologous tumor cells.

There was a significant activation of T cells after incubation with activated DCs and BCCs denoted by increased expression of CD8 on CTL by 18.5% [22.2 ± 1.7 and 26.3 ± 2 before and after interaction, respectively, *p* = 0.031], increased expression of CD4 on Th by 5.8% [49.6 ± 4.6 and 52.5 ± 4.4 before and after interaction, respectively, *p* = 0.027], and increased expression of CD103 on memory T cells by 18.5% [20.5 ± 2.1 and 24.3 ± 2.1 before and after interaction, respectively, *p* = 0.011]. While there was a significant decrease in the expression levels of CD25.CD8 by −19.6% [5.6 ± 0.9 and 4.5 ± 0.7 before and after interaction, respectively, *p* = 0.014], and there was a significant decrease in the expression level of FOXP3 by −25.1% [17.5 ± 2.8 and 13.1 ± 2.1 before and after interaction, respectively, *p* = 0.014]. Moreover, this was supported by the significant increase of the CD8/FOXP3 ratio before (2.6 ± 0.4) and after interaction (4.5 ± 0.8) by 73.1% (*p* < 0.001). The CD25 expression was decreased by −14.8% [15.5 ± 1.1 and 13.2 ± 1 before and after interaction, respectively, *p* = 0.113]. Also, FOXP3.CD103 expression was decreased by −34.5% [11.3 ± 2.4 and 8.4 ± 1.7 before and after interaction, respectively, *p* = 0.306], while there was no significant change detected in the combined expression of CD4.CD25 before (9.6 ± 1.3) and after (9.6 ± 1.2) interaction, *p* = 0.979 (Figs. 4, 5).

### Assessment of CTLA4, PD1, and FOXP3 Molecular Markers

**Figure 6:**
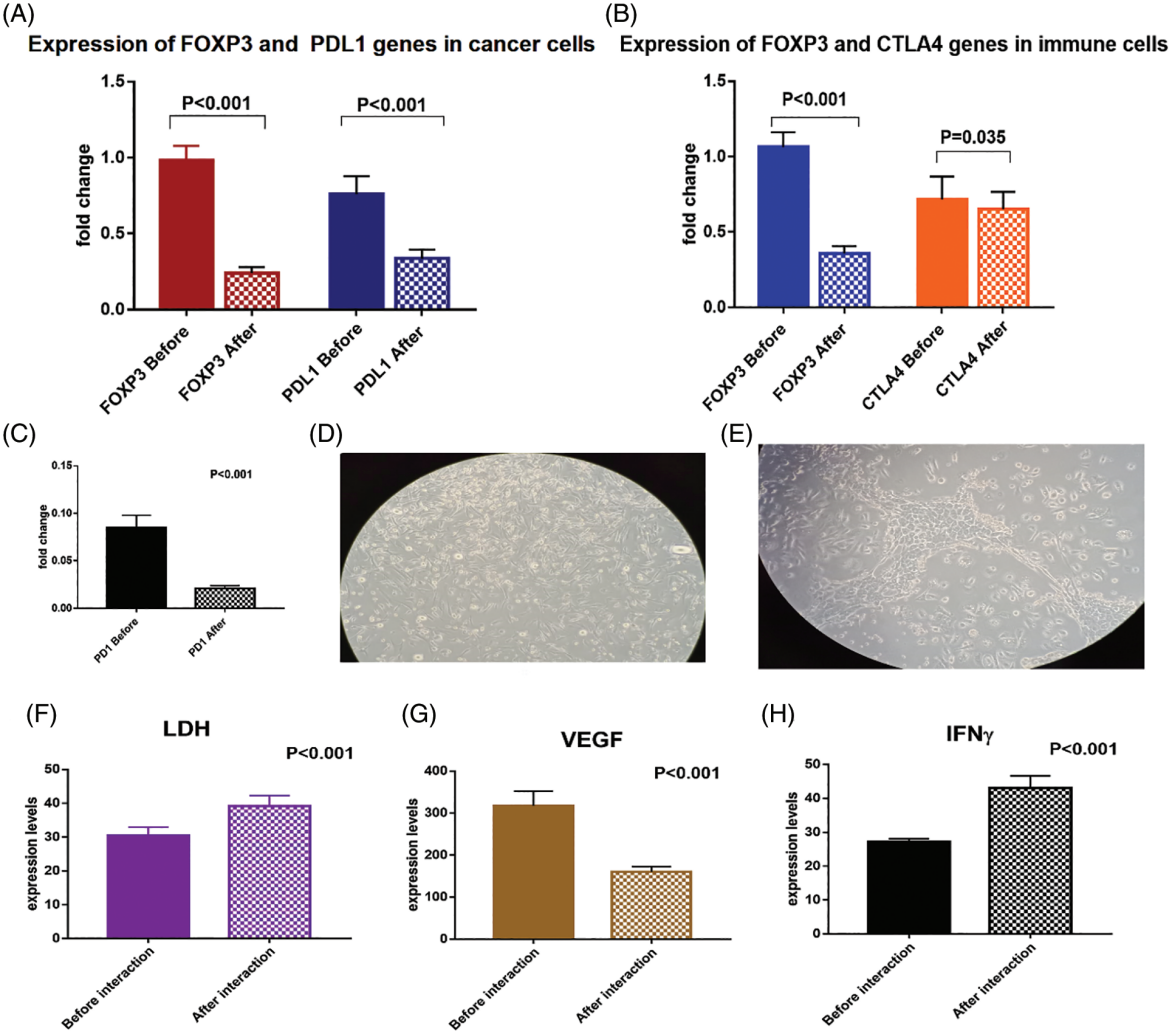
(A) Expression levels of FOXP3 and PDL1 genes in breast cancer cells before and after interaction with the dendritic cell-based vaccine, (B) expression levels of FOXP3, CTLA4 and (C) PD1 genes in immune cells before and after interaction with the breast cancer cells. (D) breast cancer cells loaded with immune cell mixture (magnification power is 20X), (E) interaction between tumor cells and immune cells after 24 h showing decreased number of tumor cells (magnification power is 20X). Expression levels of (F) LDH, (G) VEGF and (H) IFN-γ proteins in the media before and after interaction of breast cancer cells with the dendritic cell-based immunotherapy

The mRNA transcript of FOXP3 and PDL1 genes were significantly downregulated in the isolated BCCs after exposure to the immune cell mixture that was activated with checkpoint inhibitors (Fig. 6A). The FOXP3 was downregulated by −75.5% [0.98 ± 0.09 compared to 0.24 ± 0.04 before and after interaction; respectively, *p* < 0.001]. Also, PDL1 was significantly downregulated by −55.3% [0.76 ± 0.12 compared to 0.34 ± 0.06 before and after interaction, respectively, *p* < 0.001].

To confirm the activation of T cells with the checkpoint inhibitors, we assessed the expression levels of CTLA4, PD1, and FOXP3 mRNA (Figs. 6B, 6C). There was a significant downregulation of CTLA4 by −10.8% [0.72 ± 0.15 and 0.65 ± 0.11 before and after interaction; respectively, *p* = 0.035], and downregulation of PD1 by −75% [0.08 ± 0.01 and 0.02 ± 0.003 before and after interaction, respectively, *p* < 0.001]. In addition, there was a significant downregulation of FOXP3 gene expression by −66% [1.06 ± 0.09 and 0.36 ± 0.05 before and after interaction, respectively, *p* < 0.001].

### Assessment of VEGF, LDH, and IFN-γ protein levels in the culture media

The expression level of IFN-γ protein was significantly upregulated (by 58.5%) in the culture media before (27.2 ± 0.84) and after (43.1 ± 3.6) interaction of the BCCs and the immune cells activated with checkpoint inhibitors (*p* < 0.001). Moreover, LDH protein level was significantly upregulated by 28.2% [30.5 ± 2.4 and 39.1 ± 3.1, before and after interaction, respectively, *p* = 0.02], which denotes increased cytotoxicity of the activated CTL. On the other hand, the VEGF protein level was significantly downregulated by −49.5% [317.1 ± 35.1 and 160.2 ± 12, before and after interaction, respectively, *p* < 0.001, Figs. 6D–6H].

## Discussion

In the last few years, immunotherapy emerged as a promising therapeutic approach for different tumors, especially in those having a specific tumor-associated antigen including breast cancer. However, some breast cancer patients did not respond to cellular or other immunotherapy including monoclonal antibodies, checkpoint inhibitors, or oncolytic virus therapy, and consequently, still there is an increased incidence of relapse and mortality [27].

In the present study, we tried to optimize the conditions for producing an active immune response against BCCs, this was achieved through priming of the DCs with autologous BCCs. At the same time, T cells were treated with anti-CTLA4 and anti-PD1 antibodies for blocking the inhibitory signals produced by BCCs to evade immune surveillance.

In order to evaluate the efficacy of this immunotherapy, we performed many investigations to ensure its effectiveness against the BCCs. These measures included: (1) Assessment of DCs activation through the significant increase of the surface markers expression (CD86 and CD83), which leads to the production of higher levels of the Th1 effector cytokine IL12. (2) Assessment of the cytotoxic response against the tumor cells through the significant decrease of FOXP3, CD25, CD4 regulatory T cells, in addition to the significant increase in the expression of CD8 (CTL), CD4 (Th), and CD103 memory cells, which mediated killing of the BCCs through increased production of IFN-γ and LDH. On the other hand, tumor cells were evaluated for the expression of the surface markers CD133, CD44, and CD34. Our results showed a significant decrease in all these markers on tumor cells, in addition to the metastatic marker VEGF, which ensures effective tumor cell inhibition. Furthermore, there was a downregulation of gene expressions encoding FOXP3, CTLA4, PD1, and PDL1 in the cell mixture.

There are many trials performed to develop an efficient active DCs-based immunotherapy for breast cancer patients, however, their clinical benefit is still not promising. The main obstacles facing these trials are difficulties in producing broad and robust immune responses, as well as overcoming immune escape mechanisms [4,27]. Thus, in the present study, we tried to overcome these major obstacles by mixing the peripheral blood immature DCs, and naïve T cells together with TIDCs, and TILs during interaction with BCCs. This will allow for better recognition and identification of the heterogeneous and broad types of breast tumor antigens, and consequently better effective immune response. As a matter of fact, an efficient DCs-based immunotherapy depends greatly upon the ability of the DCs to be loaded with the proper tumor antigens and thereby confer long-lasting immunity [28]. This was achieved in the present study by the significant increase of CTL (CD8) by 18.5%, and the tissue-resident memory T cells (CD103) by 18.5%. These results are in agreement with many published studies reported the importance of CD103 TILS as a good prognostic factor for better relapse-free survival and overall survival in breast cancer patients [29–31].

Another method to potentiate this DCs-based immunotherapy is the treatment of the T cells with anti-CTLA4 and anti-PD1 antibodies during interaction with the autologous BCCs. CTLA4 was reported to inhibit the immune response at the initial stage of T-cells activation, while PD-1 downregulates ongoing immunological effects at sites of reaction, either in the tumor tissue or in the peripheral blood [32]. These results are supported by that of Chen et al. [4], who concluded that CTLA4 blockade in BCCs could recover the antigen-presenting function of DCs, cytokine production, and T cell activation. Also, it suppressed the biological activity and induced apoptosis of BCCs *in vitro*. Similarly, Liu et al. [33], who investigated the utility of MUC1-based mRNA vaccine, they proposed that the combination of mRNA vaccine with anti-CTLA4 mAb can induce T cell immune response significantly greater than treatment with either the mRNA vaccine or the anti-CTLA4 mAb alone. Moreover, Özverel et al. [34], reported that Mice treated with HER2/neu-loaded DCs vaccine combined with QS-21 and anti-PD-L1 mAb had significantly decreased tumor sizes, compared to mice treated with vaccine alone. In agreement with these data, Bastaki et al. [35] and Crosby et al. [36], demonstrated that combining anti-PD-1/PD-L1 therapy with immune-stimulating vaccines can be considered an effective therapeutic regimen in breast cancer.

In line with these studies, the present data showed significant downregulation of FOXP3, CTLA4, PD1, and PDL1 after the mixture of the immune cells and BCCs. As a result, this leads to inhibition of CTLA4 and PD-1 pathways, which were considered the most important immunoregulatory pathways that configure immune responses in a variety of immunological disorders including cancers and autoimmune diseases. CTLA-4 and PD-1 signaling pathways can inhibit T-cell proliferation, survival, and activation, in addition to the suppression of IFN-γ, IL-2, and tumor necrosis factor-α production [37]. Moreover, CTLA4 interacts with PKCη through CTLA4/PKCη/GIT/PAK/PIX signaling pathway in Tregs, which leads to depletion of the costimulatory molecule CD86 on intratumoral CD103+ DCs, inactivation of CD8+ T cells, and inhibition of effector cytokines production by these cells [38]. Furthermore, FOXP3 suppression can inhibit tumor invasion and metastasis via downregulating the angiogenic factor VEGF, the epithelial-mesenchymal transition (EMT), and the Notch1/Hes1 pathway [39]. Additionally, FOXP3 was reported to promote cell proliferation, invasion and EMT through activation of the Wnt signaling pathway, and consequently upregulation of β-catenin and transcription factor 4 (TCF4) [40].

Furthermore, the efficacy of our developed immunotherapy to elicit an effective CTL response and suppress Treg was evaluated through the significant increase of the CD8/FOXP3 ratio by 73.1%. These data are supported by many recently published studies demonstrated that increased CD8+/FOXP3+ ratio in breast cancer patients could be used as a good prognostic marker following neoadjuvant chemotherapy, as well as for prolonged relapse-free survival and overall survival rates compared to those with decreased CD8+/FOXP3+ ratio [41,42].

On the other hand, our developed immunotherapy was also evaluated through its influence on changing the behavior and characteristics of the cancer cells themselves. Our data revealed a significant decrease of CD44 by 22.2%, CD34 by −23.6% and CD133 by −16.9% on BCCs. CD44 was proved to be a marker of BC progression and stem-cell properties [43], and CD34 can promote tumor vascular endothelial cell proliferation, tumor invasion, and metastasis [44]. It was concluded by Zhao et al. [45], that CD34 expression together with ER, and p53 have significant roles in the incidence and development of breast cancer. Similarly, CD133 is defined as a cancer stem cell marker, it had been reported in a review done by Brugnoli et al. [46], that it can be considered a poor prognostic factor and indicator of malignant progression in breast cancer patients.

## Conclusion

The current study provided evidence that DCs based immunotherapy could be a potent and effective therapy if we used a mixture of peripheral blood DCs and T cells together with TIDCs and TILs. In addition, treating this mixture of immune cells with the immune checkpoint inhibitors (anti-CTLA4 and anti-PD1), which allow for downregulation of Tregs, proper activation of CTL, Th, and memory T cells, with subsequent suppression of tumor cells through downregulation of CD133, CD34, and CD44. However, these results are preliminary and should be validated in further studies using a larger number of samples with different types and stages of breast cancer. Also, the quality and purity of the cells should be validated rigorously for assessment in an experimental animal model, to be transferred for clinical trials.

## Data Availability

All required data and materials are available upon request, except for the personal data of the patients.
